# The Nonantibiotic Small Molecule Cyslabdan Enhances the Potency of β-Lactams against MRSA by Inhibiting Pentaglycine Interpeptide Bridge Synthesis

**DOI:** 10.1371/journal.pone.0048981

**Published:** 2012-11-06

**Authors:** Nobuhiro Koyama, Yuriko Tokura, Daniela Münch, Hans-Georg Sahl, Tanja Schneider, Yoshio Shibagaki, Haruo Ikeda, Hiroshi Tomoda

**Affiliations:** 1 Graduate School of Pharmaceutical Sciences, Kitasato University, Tokyo, Japan; 2 Institute of Medical Microbiology, University of Bonn, Bonn, Germany; 3 Kitasato Institute for Life Sciences, Kitasato University, Kanagawa, Japan; The Scripps Research Institute and Sorrento Therapeutics, Inc., United States of America

## Abstract

The nonantibiotic small molecule cyslabdan, a labdan-type diterpene produced by *Streptomyces* sp. K04-0144, markedly potentiated the activity of the β-lactam drug imipenem against methicillin-resistant *Staphylococcus aureus* (MRSA). To study the mechanism of action of cyslabdan, the proteins that bind to cyslabdan were investigated in an MRSA lysate, which led to the identification of FemA, which is involved in the synthesis of the pentaglycine interpeptide bridge of the peptidoglycan of MRSA. Furthermore, binding assay of cyslabdan to FemB and FemX with the function similar to FemA revealed that cyslabdan had an affinity for FemB but not FemX. In an enzyme-based assay, cyslabdan inhibited FemA activity, where as did not affected FemX and FemB activities. Nonglycyl and monoglycyl murein monomers were accumulated by cyslabdan in the peptidoglycan of MRSA cell walls. These findings indicated that cyslabdan primarily inhibits FemA, thereby suppressing pentaglycine interpeptide bridge synthesis. This protein is a key factor in the determination of β-lactam resistance in MRSA, and our findings provide a new strategy for combating MRSA.

## Introduction

**Figure 1:**
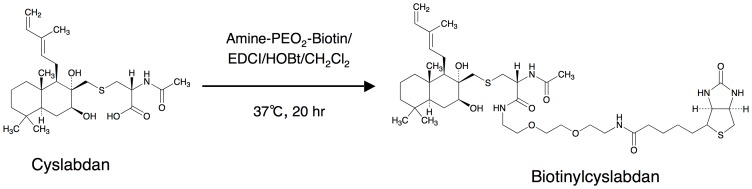
Preparation of biotinylcyslabdan. The structures of cyslabdan and biotinylcyslabdan are shown. The carboxyl moiety of the *N*-acetylcysteine residue of cyslabdan was modified with amine-(PEO)_2_-biotin to synthesize biotinylcyslabdan.

MRSA is a major nosocomial pathogen that has developed resistance to many antibiotics [1]. Moreover, it has been reported that MRSA has become resistant to the last-resort antibiotic vancomycin [2]. Therefore, it is increasingly important to find new antimicrobial agents and devise new measures that are effective against MRSA infection. Based on our screening method for new anti-infective agents [3], we have searched microbial metabolites for compounds that could restore the activity of imipenem, which is almost ineffective against MRSA. In the course of this screening program, cyslabdan (Figure 1) was isolated from the culture broth of the actinomycete strain *Streptomyces* sp. K04-0144, a soil isolate actinomycete from Ishigakijima Island, Okinawa, Japan [4], [5]. This compound consists of a unique labdan-type diterpene and an *N*-acetylcysteine residue, and enhances the activity of imipenem against MRSA by over 1,000-fold. Effect of cyslabdan on the activities of various antibiotics against MRSA is summarized in Table 1. The activities of other typical antibiotics, such as streptomycin, vancomycin, tetracycline, and ciprofloxacin, are not enhanced by cyslabdan. Among β-lactams, cyslabdan potentiated the activity of carbapenem against MRSA most effectively, i.e., by 500–1,000-fold. On the other hand, the activity of imipenem against MSSA in combination with cyslabdan was not changed, suggesting that the potentiation of cyslabdan was not effective on MSSA. As expected, a population analysis indicated that most clinical isolates of MRSA are sensitive to imipenem in combination with cyslabdan (20 µg/mL), but MSSA did not display altered minimum inhibitory concentration (MIC) values (0.03 µg/mL) for imipenem in the presence of cyslabdan. These findings prompted us to study the mechanism of action via which cyslabdan enhances the activity of imipenem against MRSA. In this study, the proteins that bind to cyslabdan were investigated in an MRSA lysate, to identify candidate target proteins that are responsible for its unique biological activity.

**Table 1:** MIC values of β–lactams and other antibiotics against MRSA in the absence and presence of cyslabdan.

	MIC (µg/mL)		
Antibiotic	None	plus cyslabdan*	Ratio; −/+
**Carbapenem**			
Imipenem	16	0.015	1067
Biapenem	16	0.03	533
Panipenem	16	0.015	1067
Meropenem	16	0.125	128
**Penam**			
Benzylpenicillin	128	16	8
Ampicillin	256	16	16
Cloxacillin	512	16	32
**Cephem**			
Cefazolin	64	8	8
Cephalexin	1024	256	4
Cefmetazole	128	4	32
Cefotaxime	1024	64	16
**Others**			
Vancomycin	0.5	0.5	1
Streptomycin	2	2	1
Tetracycline	32	32	1
Ciprofloxacin	64	64	1

* Concentration: 10 µg/mL.

## Materials and Methods

### Materials, bacterial strains, and media

Amino-PEO_2_-biotin and avidin beads (Ultralink Immobilized Streptavidin gel) were purchased from PIERCE (Rockford, IL, USA). 1-Hydroxy-1H-benzotriazole monohydrate (HOBT), 1-ethyl-3-(dimethylaminopropyl) carbodiimide (EDCI), CH_2_Cl_2_, and *N*,*N*-dimethylformamide (DMF) were purchased from KANTO Chemical (Tokyo, Japan). Isopropyl β-thiogalactopyranoside (IPTG), trypsin, iodoacetamide, dithiothreitol (DTT), mutanolysin, and sodium tetrahydroborate were purchased from SIGMA (St. Louis, MO, USA). BOCILLIN was purchased from Molecular Probes Inc. (Eugene, OR, USA). Lysostaphin, DNase, α-amylase, alkaline phosphatase, ticarcillin (TIPC), and chloramphenicol (CP) were purchased from Wako Pure Chemical Industries Ltd. (Osaka, Japan). Lysozyme was purchased from SEIKAGAKU Corp (Tokyo, Japan). TOC-39 and a clinical MRSA isolate were kindly provided by H. Hanaki (Kitasato University, Tokyo, Japan) [6]. *S. aureus* FDA209P stocks from our laboratory were used. Mueller-Hinton broth (MHB), LB broth, and LA (all DIFCO, Becton Dickinson, Sparks, MD, USA) were used to encourage the growth of the microorganisms.

### General procedure

FAB-MS spectra were recorded on a JMS-DX300 mass spectrometer (JEOL Ltd., Tokyo, Japan), and HRFAB-MS spectra were recorded on a JMS-AX505 HA mass spectrometer (JEOL Ltd., Tokyo, Japan). ESI-MS data were obtained using an Agilent 1200 Series LC system (Agilent Technologies, Palo Alto, CA, USA) and a JMS-T100LP MS system (JEOL Ltd., Tokyo, Japan). To obtain MS/MS data for the peptide fragments after in-gel trypsin digestion, a DiNa Nano-LC system (KYA Technologies, Tokyo, Japan) and a QSTAR Elite Hybrid LC-MS/MS system (Applied Biosystems, Foster City, CA, USA) were used. The peptides were loaded onto a C18 reversed-phase column (0.1×50 mm) and analyzed by linear gradient elution from 5% B (80% aqueous acetonitrile containing 0.1% formic acid) to 45% B over 30 min using solvent A (2% aqueous acetonitrile containing 0.1% formic acid) and solvent B. MS/MS spectra were analyzed using the Protein Pilot software (Applied Biosystems, Foster City, CA, USA). UV spectra were recorded on a spectrophotometer (8453, Agilent Technologies, Palo Alto, CA, USA).

### Isolation of cyslabdan

*Streptomyces* sp. K04-0144 was used to produce cyslabdan. The compound was isolated from the fermentation broth of this strain, as reported previously [4], [5].

### Preparation of biotinylcyslabdan

Biotinylcyslabdan was synthesized by modifying the carboxyl group in the *N*-acetylcysteine residue of cyslabdan. Amino-PEO_2_-biotin (21 µmol), HOBT (21 µmol), and EDCI (21 µmol) were added to a solution of cyslabdan (21 µmol) in CH_2_Cl_2_ (2.5 mL). After stirring at 37°C for 20 h, the mixture was concentrated to dryness and then treated with distilled water (3.0 mL), to stop the reaction. The reaction product was then purified by HPLC (column, PEGASIL ODS (Senshu Sci. Corp., Tokyo, Japan), 20×250 mm; solvent, 65% aqueous acetonitrile containing 0.05% trifluoroacetic acid; detection, UV at 210 nm; flow rate, 6.0 mL/min). Under these conditions, biotinylcyslabdan was eluted as a peak with a retention time of 14.7 min (Figure S1). This peak was collected and concentrated *in vacuo* to give a white powder (4.0 µmol; yield, 18.7%). The synthesis of biotinylcyslabdan was confirmed based on its physicochemical properties (Table S1).

### Assay of the potentiation of the activity of imipenem against MRSA by cyslabdan

The potentiating effects of cyslabdan on the activity of imipenem against MRSA were investigated according to our established liquid microdilution method [5].

### Preparation of an MRSA lysate

An MRSA lysate was prepared according to Malouin's method [7], with some modifications. MRSA was grown at 37°C in LB until the late exponential phase and then collected by centrifugation at 8,000 rpm for 15 min at 4°C. MRSA cells (wet weight, ∼2.0 g) were washed and resuspended in 10 mM phosphate potassium buffer (pH 7.0) containing 10 mM MgCl_2_ (32 mL) and then treated at 30°C for 30 min with lysostaphin and DNase (final concentration of each compound, 100 µg/mL), to lyse the cells. After removal of intact cells by centrifugation at 6,500 rpm for 20 min at 4°C, the supernatant was recentrifuged at 40,000 rpm for 30 min at 4°C, to yield a pale-yellow precipitate. Subsequently, protein concentration was adjusted to 5.0 mg/mL by adding the buffer described above, and the resultant MRSA lysate solution was stored at −80°C until use.

### PBP2′ binding assay

The PBP2′ binding assay was carried out using fluorescent penicillin (BOCILLIN) [7], [8]. An MRSA lysate (15 µg) in 10 mM phosphate potassium buffer (pH 7.0) containing 10 mM MgCl_2_ was preincubated with clavulanic acid (final concentration, 500 µg/mL) for 10 min at 35°C, to mask PBP other than PBP2′. Subsequently, cyslabdan or TOC-39 [6] was added to the reaction solution. After incubation for 10 min at 35°C, PBP2′ was labeled by adding 20 µM BOCILLIN and incubating the mixture for a further 10 min at 35°C. The proteins were then separated by 10% SDS polyacrylamide gel electrophoresis (SDS–PAGE) and the BOCILLIN bound to PBP2′ was detected on a fluorescent image analyzer (excitation, 488 nm; emission, 530 nm; FLA-7000, FUJIFILM, Tokyo, Japan).

### PBP2′ expression assay

The PBP2′ expression assay was carried out using an MRSA screening kit (Denka Seiken Co. Ltd., Tokyo, Japan), as reported previously by Zhao et al. [9]. MRSA was grown in MHB at 37°C until the middle exponential phase and then treated with cyslabdan (100 µg/mL) in combination with imipenem (100 µg/mL) at 37°C for 2 h. MRSA was collected by centrifugation at 8,000 rpm for 15 min at 4°C and then suspended in 10 mM phosphate potassium buffer (pH 7.0) containing 10 mM MgCl_2_. Subsequently, the PBP2′ protein was extracted from MRSA by disrupting the cells with an ultrasonicator (Bioruptor, COSMOBIO, Tokyo, Japan). The resultant extract was centrifuged at 12,000 rpm for 10 min at 4°C, to separate the supernatant containing the PBP2′ protein and unbroken cells. The protein concentration of the supernatant was determined via the Bradford method using bovine serum albumin as a standard. Each sample was adjusted to produce a protein concentration of 0.2 mg/mL and was then diluted serially by 128-fold with the buffer described above. Subsequently, these diluted samples were mixed with anti-PBP2′-monoclonal-antibody-sensitized latex. The level of PBP2′ protein production in MRSA was estimated by observing the intensity of agglutination of each sample. Furthermore, to investigate whether intact PBP2′ was expressed in MRSA, the remaining supernatant was used to analyze the reactivity between the PBP2′ protein and BOCILLIN, according to the method described above.

### Analysis of cyslabdan-binding proteins in an MRSA lysate

Biotinylcyslabdan (12 nmol) was attached to avidin beads (4.2 nmol) [10], followed by application of MRSA lysate (0.2 mg/300 µL) to the beads, which were mixed well for 1 h at 4°C with a rotator. The beads were collected by centrifugation at 3,000 rpm for 5 min at 4°C and washed three times by adding PBS (300 µL). Finally, the beads were boiled for 5 min in the presence of Laemmli sample buffer (30 µL), to recover the bound proteins. The bound proteins (20 µL) were then separated by SDS–PAGE (12.5% gel) at a constant current of 20 mA for 1.5 h and detected via silver staining.

### Identification of cyslabdan-binding proteins by LC-MS/MS

Bands from SDS–PAGE were excised and prepared for LC-MS/MS analysis, as described previously [11]. Each band was destained by washing it twice in 25 mM NH_4_HCO_3_ and acetonitrile (20 µL each) for 10 min, dehydrated with acetonitrile (40 µL) for 5 min, and then dried *in vacuo*. Reduction was performed using 10 mM DTT and 100 mM NH_4_HCO_3_ (40 µL) for 30 min at 56°C. After removing the solution, the samples were treated with 100 mM NH_4_HCO_3_ buffer containing 55 mM iodoacetamide (40 µL) and incubated for 30 min in the dark, to perform the alkylation. The samples were washed with 25 mM NH_4_HCO_3_ and acetonitrile (20 µL each) for 10 min, dehydrated with acetonitrile (40 µL) for 5 min, and dried *in vacuo*. Each band was rehydrated in 50 mM NH_4_HCO_3_ buffer containing trypsin (10 ng/µL) on ice for 45 min. The remaining trypsin was then removed and replaced with 50 mM NH_4_HCO_3_ buffer (30 µL) before incubation at 37°C for 20 h, for in-gel digestion. First, the reaction solutions containing peptide fragments were transferred into new tubes and extracted via the addition of 33% aqueous acetonitrile containing 0.1% TFA. Subsequently, an additional extraction step was performed using 70% aqueous acetonitrile containing 0.1% TFA. The extracted solutions were collected and combined with the tubes mentioned above. The peptide fragments were then analyzed by LC-MS/MS. A control band from the same gel was also analyzed, for comparison purposes. As SAR1388 exhibited the highest amino acid cover rate (56%), with a band that displayed the most significant difference in its total recovery ratio compared with the control band (sample/control: 10.1), it was selected as a candidate.

### Preparation of recombinant FemA and its related proteins

The recombinant FemA and its related proteins were prepared according to Roher's method [12], with some modifications. Briefly, genomic DNA prepared from *S. aureus* was used to amplify the *femA* gene, the *femX* gene and the *femB* gene by PCR. The PCR primers were as follows: Fwd for the *femA* gene, CGAGCTAGCAAGTTTACAAATTTAACA; Rev for the *femA* gene, GCACTCGAGAAAAATTCTGTCTTTAACT; Fwd for the *femX* gene, CGAGCTAGCGAAAAGATGCATATCACTAATC; Rev for the *femX* gene, GCACTCGAGTTTTCGTTTTAATTTACGAGATATT; Fwd for the *femB* gene, CGAGCTAGCAAATTTACAGAGTTAACTGTTAC; Rev for the *femB* gene, GCACTCGAGTTTCTTTAATTTTTTACGTAATTTATCC. The restriction site was *Nhe*I for Fwd primers, while that was *Xho*I for Rev primers. The amplified fragments were cloned into the pET21a(+) vector. The identity of the gene was confirmed by analyzing its DNA sequence. The constructed plasmid was transformed into *Escherichia coli* Rosetta (pLys) cells, allowing the expression of proteins with a C-terminal hexahistidine tag. Single colonies of this expression construct were isolated from freshly streaked LA supplemented with TIPC (final concentration, 100 µg/mL) and CP (final concentration, 30 µg/mL) and used to inoculate LB broth (10 mL) containing the antibiotics described above, before being grown overnight at 37°C and 180 rpm. A fraction (2 mL) of this culture was transferred to LB broth (200 mL) containing TIPC (final concentration, 100 µg/mL) and CP (final concentration, 30 µg/mL) and cultured at 37°C and 180 rpm until it reached an optical density of 0.6 at 600 nm. After the addition of IPTG (final concentration, 0.1 mM), the culture was incubated overnight at 24°C and 150 rpm, to induce the expression of proteins. The cells were then harvested by centrifugation at 8,000 rpm for 10 min at 4°C and washed with 50 mM sodium phosphate buffer (pH 7.5) containing 300 mM NaCl and 20% glycerol (10 mL). The cells were suspended in the same buffer, containing APMSF (final concentration, 0.1 mM) and lysozyme (final concentration, 1 mg/mL), and then incubated on ice for 30 min. After the cells had been disrupted with an ultrasonicator, the solution was centrifuged at 20,000 rpm for 30 min at 4°C, to precipitate cell debris. After the supernatant had been filtrated through a filter membrane with a pore size of 0.45 µm, the filtered sample was purified via a conventional method using a Ni column and kept for the experiment described below.

### Confirmation of the interaction between cyslabdan and recombinant FemA or its related proteins

As described in the “Analysis of cyslabdan-binding proteins in an MRSA lysate” section of Materials and Methods, biotinylcyslabdan-immobilized avidin beads were used for this analysis. Firstly, proteins (0.05 nmol/300 µL) dissolved in 50 mM sodium phosphate buffer (pH 8.0) containing 1 mM EDTA, 1 mM DTT, 20% glycerol, and NP-40 (final concentration, 0.1%) was applied to the beads, which were then mixed well for 1 h at 4°C with a rotator. Subsequently, the beads were collected by centrifugation at 3,000 rpm for 5 min at 4°C and washed three times by adding PBS (300 µL) containing NP-40 (final concentration, 0.1%). Finally, the beads were boiled for 5 min in the presence of Laemmli sample buffer (30 µL), to recover the bound proteins. The bound proteins (20 µL for FemA and FemB, and 2 µL for FemX) were analyzed using the conditions described above.

### Synthesis and purification of lipid II, monoglycyl lipid II and triglycyl lipid II

Lipid II was prepared by reacting undecaprenyl phosphate, UDP-MurNAc-pentapeptide, GlcNAc and membrane proteins of *Micrococcus luteus* as described previously [13], [14]. Glycine-labeled lipid intermediates, monoglycyl lipid II and triglycyl lipid II were prepared by reacting recombinant FemX and FemA proteins and their purified substrates, lipid II and monoglycyl lipid II, respectively [14]. Synthesized lipid intermediates were extracted from the reaction mixtures with an equal volume of butanol/pyridine acetate (2∶1; vol∶vol; pH 4.2). To purify lipid intermediates, the extract samples were subjected to a DEAE-cellulose column and eluted in a linear gradient from chloroform–methanol–water (2∶3∶1) to chloroform–methanol-300 mM ammonium bicarbonate (2∶3∶1). Fractions containing lipid intermediates were identified by TLC analysis using the developing solvent of chloroform–methanol–water–ammonia (88∶48∶10∶1) [15]. The concentration of purified lipids was calculated by measuring inorganic phosphates released after the treatment with perchloric acid [16].

### Assay for the enzymatic activity of FemX, FemA and FemB proteins

The assay for the enzymatic activity of FemX, FemA and FemB were carried out as described previously [14]. Briefly, the reaction solutions were adjusted to be a total volume of 100 µl containing 2.5 nmol of purified lipid intermediate (lipid II, monoglycyl lipid II or triglycyl lipid II), 10 µg of glycyl-tRNA-synthetase and 25 µg of tRNA, 2 mM ATP and 50 nmol of [^14^C]-glycine in 100 mM Tris-HCl, 20 mM MgCl_2_, pH 7.5, and 0.8% Triton X-100. Then, purified FemX, FemA and FemB proteins were added to the corresponding reaction solutions at a concentration of 2.7 µg, and the reaction solutions were incubated for 90 min at 30°C in the presence and absence of cyslabdan (0.8 mM). After that, lipid intermediates were extracted from the reaction mixtures and analyzed by TLC using the developing solvent of chloroform–methanol–water–ammonia (88∶48∶10∶1). Finally, the amount of monoglycyl lipid II, triglycyl lipid II and pentgalycyl lipid II was quantified using phosphoimaging in a Storm™ imaging system (GE Healthcare, Freiburg, Germany). For determination of IC_50_ value, the concentration of cyslabdan was used in the range of 0 to 1.2 mM. The IC_50_ value represents the average of three independent experiments.

### HPLC analysis of the composition of the peptidoglycan of cyslabdan-treated MRSA

HPLC analysis of the composition of MRSA peptidoglycans was carried out according to the established method [17]. Briefly, MRSA was cultured in the presence or absence of cyslabdan (4 µg/mL). The cells were then treated with various enzymes (α-amylase, DNase, RNase, trypsin, and alkaline phosphatase) to prepare peptidoglycan samples. Subsequently, peptidoglycans were hydrolyzed with mutanolysin and reduced with sodium tetrahydroborate to form muropeptides. The resultant products were analyzed by HPLC (column, YMC-Triart C18 4.6×250 mm (YMC Co., Ltd., Kyoto, Japan); detection, UV 206 mm; flow rate, 0.8 mL/min; mobile phase, linear gradient elution from 0 to 5% solvent over 20 min, and from 5 to 30% solvent over 150 min using a methanol solution containing 100 mM sodium phosphate buffer as a solvent). Consistent with the previous data reported by de Jonge et al. [17], monomer-, dimer-, trimer-, tetramer-, and oligomer-type mureins were detected. As the accumulation of muropeptides was observed in the monomeric fraction of cyslabdan-treated MRSA, we analyzed mainly this fraction. To identify these muropeptides, the samples were collected by preparative HPLC under the same conditions and were then desalted with a Sep-Pak cartridge (Waters, Milford, MA, USA) and analyzed via ESI-MS. The muropeptides identified were as follows: peak 1 (nonglycyl muropeptide), [M+2Na-H]^+^ = 1012.5; peak 2 (monoglycyl muropeptide), [M+2Na-H]^+^ = 1069.6; and peak 3 (pentaglycyl muropeptide), [M+Na]^+^ = 1275.7 (Figure S2).

## Results

### Effects of cyslabdan on the resistance mechanism of MRSA

MRSA possesses a β-lactam-insensitive transpeptidase (PBP2′) and/or β-lactamases as their major resistance mechanism [18], [19]. Several compounds have been reported to enhance the activity of β-lactam against MRSA (Figure S3): polyphenols (epigallocatechin gallate, corilagin, and tellimagrandin I) [8], [9] from plants; diterpene totarol from the totara tree [20]; and the synthetic compound MC-200,616 (presented at the 35th meeting of Interscience Conference on Antimicrobial Agents and Chemotherapy (1995)). A mechanistic study suggested that these compounds affect the expression and/or function of PBP2′ in MRSA. As cyslabdan is structurally related to totarol and MC-200,616, the effects of the compound on the function and expression of PBP2′ were investigated. Although it fully potentiated the activity of imipenem against MRSA at 10 µg/mL, cyslabdan did not inhibit the binding of fluorescent penicillin to PBP2′ or the expression of PBP2′ in MRSA, even at 1000 µg/mL, indicating that cyslabdan has no effect on PBP2′ (Figures S4 and S5). Furthermore, cyslabdan had no effect on β-lactamase (penicillinase or cephalosporinase) activity in MRSA [5], even at 100 µg/mL. These findings strongly suggest that the imipenem-potentiating activity of cyslabdan occurs via different mechanisms.

### Analysis of cyslabdan-binding proteins in an MRSA lysate

**Figure 2:**
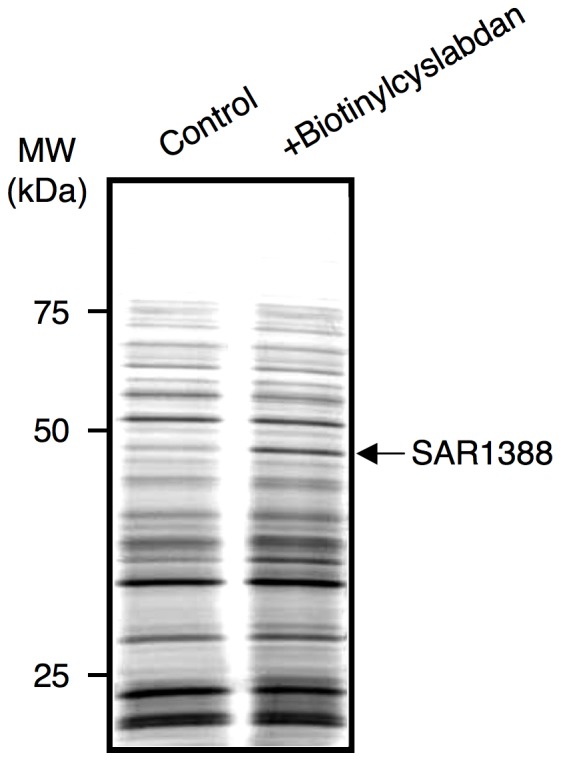
Identification of cyslabdan-binding proteins. Biotinylcyslabdan was attached to avidin beads and incubated with an MRSA lysate. After washing nonspecific proteins with PBS, the bound proteins were recovered from the beads via denaturation with Laemmli sample buffer and were then analyzed via SDS–PAGE. Typical data from five separate experiments are shown. The arrow indicates a band around 50 kDa that was detected reproducibly.

To examine the mechanism of action of cyslabdan, we employed a different strategy: i.e., the identification of cyslabdan-binding proteins in an MRSA lysate. To do this, biotinylcyslabdan (Figure 1) was prepared by modifying the carboxyl moiety of the *N*-acetylcysteine residue of cyslabdan. After checking that it retained its imipenem-potentiating activity against MRSA (Table S2), biotinylcyslabdan was attached to avidin beads. Then, a lysate prepared from MRSA was applied to the beads. After washing the beads with PBS, the bound proteins were recovered via denaturation with Laemmli sample buffer and were analyzed by SDS–PAGE. As a result, one protein band around 50 kDa was reproducibly detected and identified as SAR1388 by LC-MS/MS (Figure 2).

### Examination of the binding of cyslabdan to SAR1388 and its related proteins

**Figure 3:**
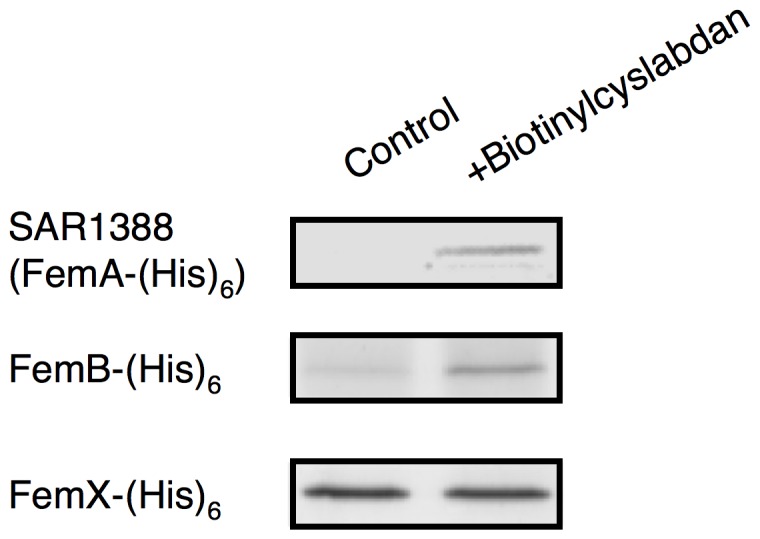
Binding of cyslabdan to hexahistidine-tagged SAR1388 and its related proteins. Biotinylcyslabdan-immobilized avidin beads were used to analyze the interaction between cyslabdan and the proteins of FemA, FemX and FemB. The bound proteins were recovered by denaturing with Laemmli sample buffer and were analyzed using SDS–PAGE.

The binding of SAR1388 to cyslabdan was confirmed as follows. Hexahistidine-tagged SAR1388 protein was obtained by expression in *E. coli*. Purified recombinant SAR1388 protein was then added to avidin beads onto which biotinylcyslabdan had been immobilized. Subsequently, the protein that bound to cyslabdan was recovered in the manner described above and identified as SAR1388 (Figure 3). This result indicated that cyslabdan has affinity for SAR1388.

SAR1388 is also known as FemA, which was reported to be involved in the synthesis of peptidoglycan in MRSA [21]–[23]. MRSA possesses a unique pentaglycine interpeptide bridge between the murein monomers in its peptidoglycan molecule. Four enzymes are involved in the formation of this bridge: FemX works to add the first Gly from the l-Lys of the murein monomer; FemA adds the second and third Gly residues; FemB adds the fourth and fifth Gly residues; and PBP and/or PBP2′ cross-link the terminal Gly of the pentaglycine region of the murein monomer to the fourth d-Ala of the pentapeptide in the next murein monomer to construct the MRSA peptidoglycan. A *femA*-deficient MRSA mutant was reported to display susceptibility to β-lactam drugs [23], [24], strongly suggesting that FemA is the molecular target of cyslabdan. On the other hand, FemA is functionally related to FemB and FemX. Therefore, the binding of cyslabdan to these proteins was studied using recombinant FemX and FemB. As shown in Figure 3, FemB was bound to only avidin beads, while the binding amount of FemB increased in the presence of biotinylcyslabdan. The binding amount of FemX in the presence of biotilylcyslabdan was not changed compared with control, suggesting that cyslabdan has affinity for FemB but not FemX.

### Effects of cyslabdan on enzymatic activity of FemA and its related proteins

**Figure 4:**
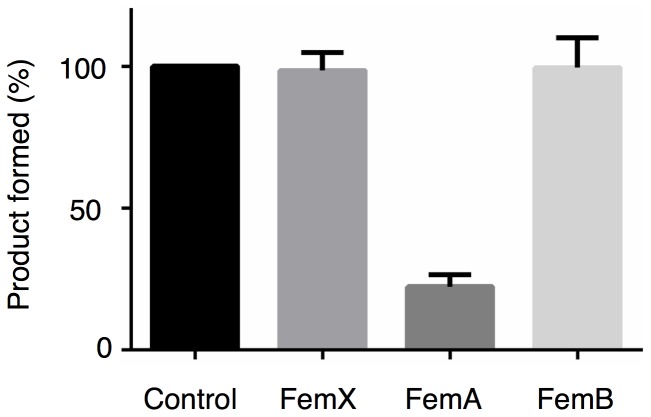
Effect of cyslabdan on FemX, FemA and FemB activities. The amount of monoglycyl lipid II, triglycyl lipid II and pentaglycyl lipid II produced by FemX-, FemA- and FemB-catalyzed reaction was compared in the presence and absence of cyslabdan (0.8 mM). The amount of each product formed in the absence of cyslabdan is set up at 100%, and each product formed in the presence of cyslabdan is expressed as a percentage of control.

To identify the molecular target of cyslabdan, its impact on the individual FemX, FemA and FemB reactions was investigated *in vitro* using purified proteins and their respective substrates. In the presence of cyslabdan (0.8 mM), the enzymatic activity of FemA, catalyzing the conversion of monoglycyl lipid II to triglycyl lipid II, was almost completely inhibited, whereas the FemX and FemB reactions were unaffected (Figure 4). Furthermore, the FemA-catalyzed reaction was dose-dependently inhibited by cyslabdan with an IC_50_ of 0.53 mM. These results indicated that cyslabdan primarily targets FemA.

### Effects of cyslabdan on the peptidoglycan of the cell wall of MRSA

**Figure 5:**
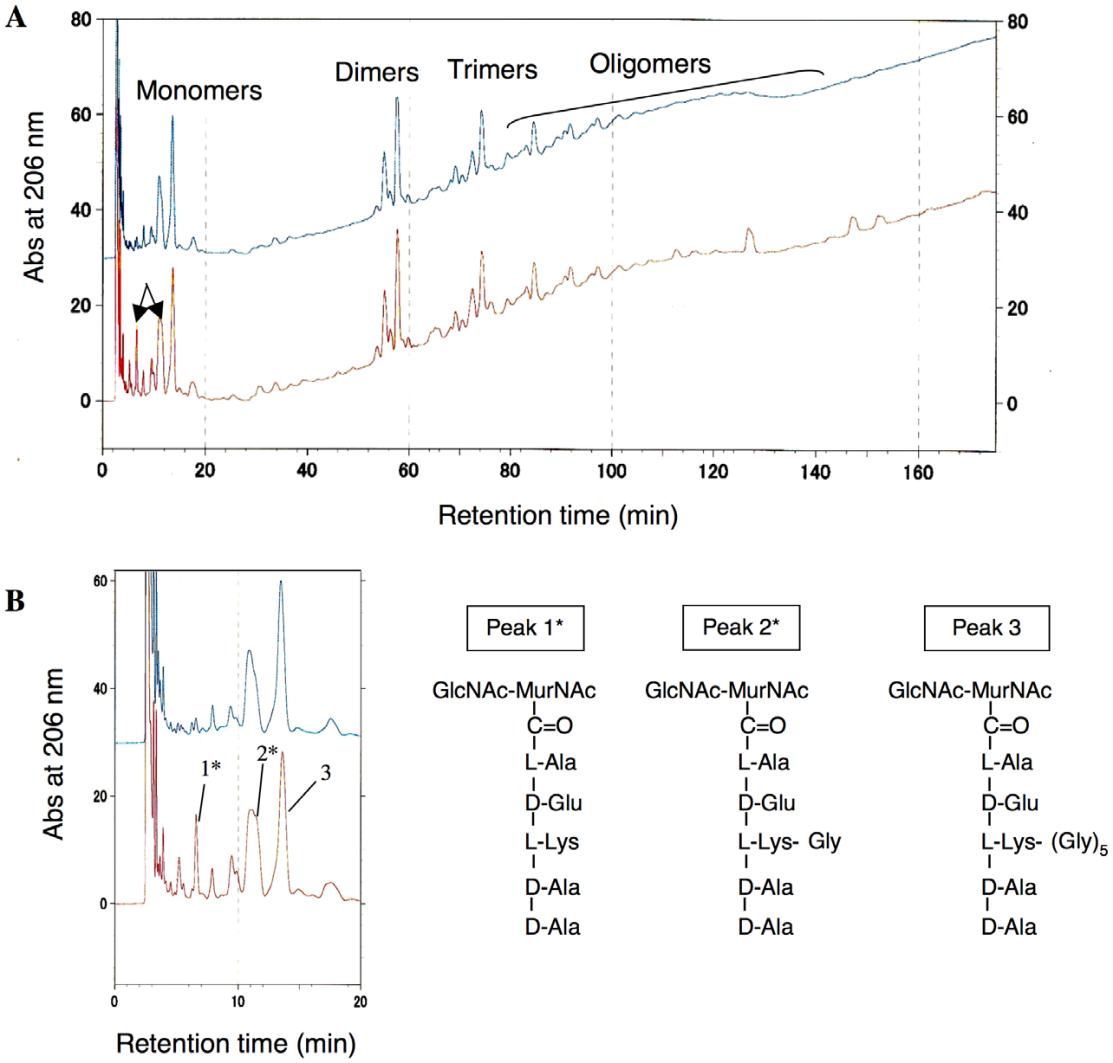
HPLC analysis of the peptidoglycan composition of cyslabdan-treated MRSA. (A) HPLC analysis of the muropeptides extracted from MRSA peptidoglycans. Peptidoglycans were prepared from MRSA in the presence or absence of cyslabdan (4 µg/mL). After treating the peptidoglycans with mutanolysin and sodium tetrahydroborate, the resultant monomer-, dimer-, trimer-, and oligomer-type muropeptide products were analyzed by HPLC under the established conditions using an ODS column. The upper chromatogram indicates the results obtained for control MRSA, whereas the lower chromatogram indicates those obtained for cyslabdan-treated MRSA. (B) Structures of monomer-type muropeptides identified by ESI-MS. The HPLC results obtained for the monomeric fraction have been enlarged on the left-hand side. Peaks 1–3 were recovered and analyzed by ESI-MS. The structures of peaks 1–3 are shown on the right-hand side. Peaks 1 and 2 (asterisks) accumulated in the cyslabdan-treated MRSA.

To confirm whether cyslabdan acts on the peptidoglycan of the cell wall of MRSA, the structures of MRSA peptidoglycans were compared in the presence and absence of cyslabdan (4 µg/mL). MRSA peptidoglycans were obtained and then treated with mutanolysin and sodium tetraborohydrate, and the various types of murein products were analyzed by HPLC under the established conditions using an ODS column [17]. As shown in Figure 5A, monomer-, dimer-, trimer-, tetramer-, and oligomer-type mureins were detected. In particular, two peaks (peaks 1 and 2) in the murein monomer fraction were increased in the peptidoglycans recovered from the cyslabdan-treated MRSA. These structures were identified as monoglycyl and nonglycyl murein monomers by mass spectrum analysis (Figure 5B). In this fraction, the main peak represented a pentaglycyl murein monomer (peak 3), whereas no triglycyl murein monomers were observed in the presence of cyslabdan (Figure 5B). Actually, a *femA*-deficient MRSA mutant was reported to accumulate nonglycyl murein monomers together with monoglycyl murein monomers [23], which were very similar to our results. Taken together, these results led us to conclude that cyslabdan primarily inhibits FemA, thereby blocking pentaglycine interpeptide bridge synthesis.

## Discussion

**Figure 6:**
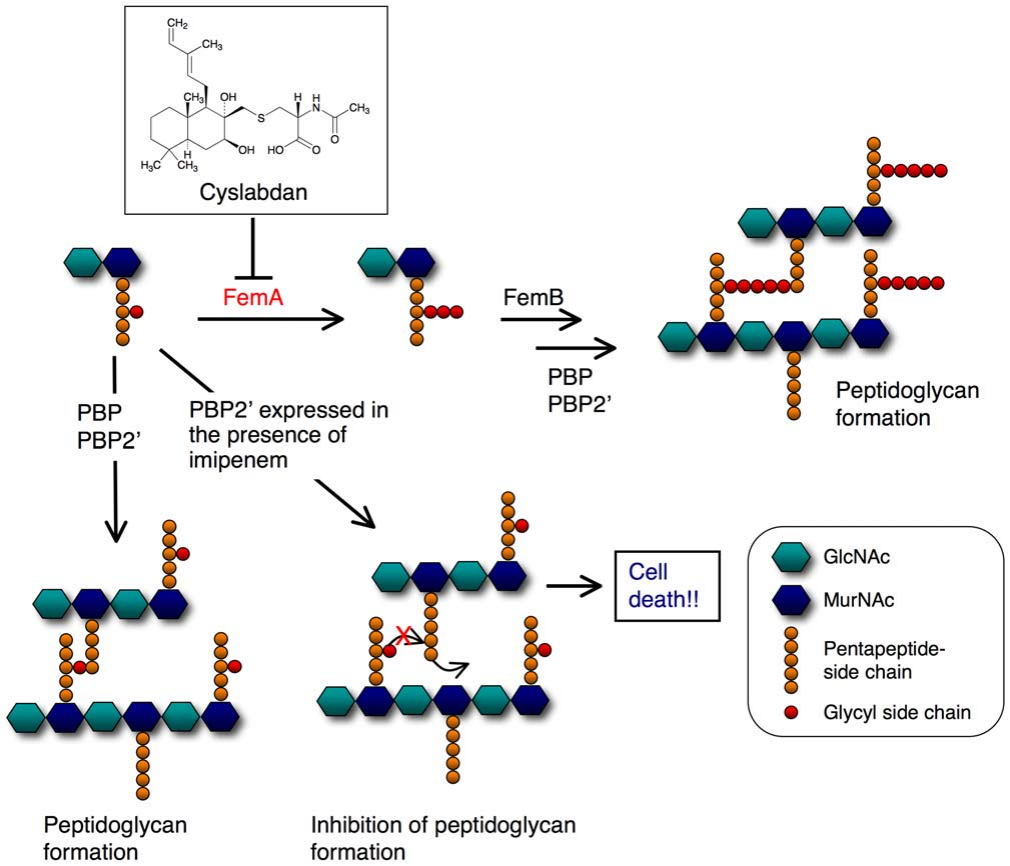
Proposed mechanism underlying the effect of cyslabdan on the activity of imipenem against MRSA. In MRSA, the first Gly is extended from the l-Lys of the pentapeptide region of the murein monomer (MurNAc-GlcNAc-pentapeptide) by FemX. The introduction of the second and third Gly is catalyzed by FemA, and the fourth and fifth Gly are added by FemB, to form (Gly)_5_-murein monomers. PBP and/or PBP2′ finally catalyze the cross-linking of the fifth Gly and fourth d-Ala of the pentapeptide region in the next murein monomer together with the concomitant release of the terminal d-Ala of the pentapeptide. Imipenem alone: β-lactam inhibited PBP, but insensitive PBP2′ was able to cross-link (Gly)_5_-murein monomers. Therefore, MRSA can grow even in the presence of imipenem. Cyslabdan alone: the nonantibiotic compound cyslabdan inhibited FemA, causing the accumulation of Gly-murein monomers, but PBP and/or PBP2′ were able to cross-link them; hence, MRSA can grow even in the presence of cyslabdan. Combination of imipenem and cyslabdan: MRSA was not able to grow, indicating that PBP2′ cannot cross-link Gly-murein monomers. In this illustration, the red cross indicates the observation that PBP2′ was not able to cross-link the resulting monoglycyl murein monomer to the next murein monomer.

Our proposed mechanism for the potentiation of the activity of imipenem against MRSA by cyslabdan is illustrated in Figure 6. Treatment of MRSA with cyslabdan led to the inhibition of FemA, which resulted in the accumulation of monoglycyl or nonglycyl murein monomers. However, cyslabdan itself had almost no effect on the growth of MRSA, indicating that PBP and/or PBP2′ recognize the monoglycyl murein monomers as a substrate and cross-link them to construct peptidoglycans. In agreement with our findings, a *femA*-deficient MRSA mutant was reported to be able to grow as efficiently as wild strains of MRSA [23]. Conversely, treatment with a combination of imipenem and cyslabdan completely inhibited the growth of MRSA, indicating that imipenem-insensitive PBP2′ cannot cross-link monoglycyl murein monomers, resulting in the failure of MRSA peptidoglycan formation.

Very recently, Ejim et al. reported that combinations of antibiotic and nonantibiotic compounds displayed enhanced antimicrobial efficacy in their *Pseudomonas aeruginosa* model [24]. Nonantibiotic compounds are considered to have potential as new anti-infectious agents. Furthermore, Huber et al. recently reported that two series of small indole molecules potentiate the activity of the β-lactam drug imipenem against MRSA [25]. Interestingly, a chemical genetic study revealed that these compounds target SAV1754, a homolog of *E. coli* flippase, which is involved in peptidoglycan synthesis. SAV1754 is predicted to display a similar function in MRSA. SAV1754 is thought to be essential for bacterial growth, but might be unrelated to the target of cyslabdan, as cyslabdan alone did not suppress the growth of MRSA.

In summary, proteins that bind to cyslabdan were investigated in an MRSA lysate, which led to the identification of FemA, the function of which was predicted by genetic studies to be involved in pentaglycine interpeptide bridge formation [23]. Actually, cyslabdan inhibited the addition reaction of glycine catalyzed by FemA in an enzyme-based assay. The inhibitory activity was very weak, but this might be due to low sensitivity of this enzymatic assay system. Furthermore, it was suggested that cyslabdan has affinity for FemB as well as FemA. This result is plausible because FemA and FemB share high homology and similar function. However, the enzymatic activity of FemB was unaffected by cyslabdan. In addition, FemB was not detected as the proteins that bind to cyslabdan in an MRSA lysate. These results suggested that cyslabdan is bound to FemB with a binding mode different from FemA, and probably its affinity for FemB is weaker than FemA. Taken together, it led us to conclude that a primary molecular target of cyslabdan is FemA. No small molecules that target FemA have been reported to date. To the best of our knowledge, cyslabdan is the first known FemA inhibitor. The accumulation of monomeric murein monomers in cysladan-treated MRSA peptioglycan is very similar to previous report using a *femA*-deficient MRSA mutant [23]. Specifically, one important finding of this study was that PBP2′ was not able to cross-link monoglycyl and nonglycyl murein monomers to form peptidoglycans in MRSA. PBP2′ might only recognize pentaglycine murein monomers during peptidoglycan formation in MRSA. Therefore, the biosynthetic pathway for the pentaglycine interpeptide bridge is a potential target for enhancing the potency of β-lactam drugs that are ineffective against MRSA. Small molecule inhibitors could be promising candidates for combination therapies to combat MRSA. Furthermore, such inhibitors could serve as chemical tools for investigating the molecular mechanisms of the synthesis of the peptidoglycan of the cell walls of MRSA.

## Supporting Information

Figure S1 **Purification of biotinylcyslabdan via HPLC.**
(TIF)

Figure S2 **ESI-MS spectrum of the muropeptides identified in the monomeric region of the cell wall of MRSA.**
(TIF)

Figure S3 **Structures of reported imipenem potentiators.**
(TIF)

Figure S4 **Effect of cyslabdan on PBP2′ binding in MRSA.** An MRSA lysate was preincubated with clavulanic acid to mask PBPs other than PBP2′. The sample was treated with TOC-39 (10–1,000 µg/mL, upper panel) or cyslabdan (250–1,000 µg/mL, lower panel), followed by the labeling of PBP2′ with the fluorescent penicillin BOCILLIN. The samples were analyzed by SDS–PAGE. The arrow indicates the position of PBP2′.
(TIF)

Figure S5 **Effect of cyslabdan on PBP2′ expression in MRSA.** PBP2′ proteins were extracted from MRSA treated with methanol solvent (lane 1), imipenem alone (lane 2), cyslabdan alone (lane 3), and cyslabdan plus imipenem (lane 4) and analyzed via SDS–PAGE using BOCILLIN. Concomitantly, the remaining sample was used to perform a latex agglutination test using an anti-PBP2′ antibody. All samples exhibited the same titer of agglutination (×1/32).
(TIF)

Table S1 **Physicochemical properties of biotinylcyslabdan.**
(TIF)

Table S2 **Imipenem-potentiating activity of biotinylcyslabdan against MRSA.**
(TIF)
