# Supplementary figures and images for: The Nonantibiotic Small Molecule Cyslabdan Enhances the Potency of β-Lactams against MRSA by Inhibiting Pentaglycine Interpeptide Bridge Synthesis
Source: PLoS One. 2012 Nov 6;7(11):e48981. doi: 10.1371/journal.pone.0048981 (PMC3490914; doi:10.1371/journal.pone.0048981)

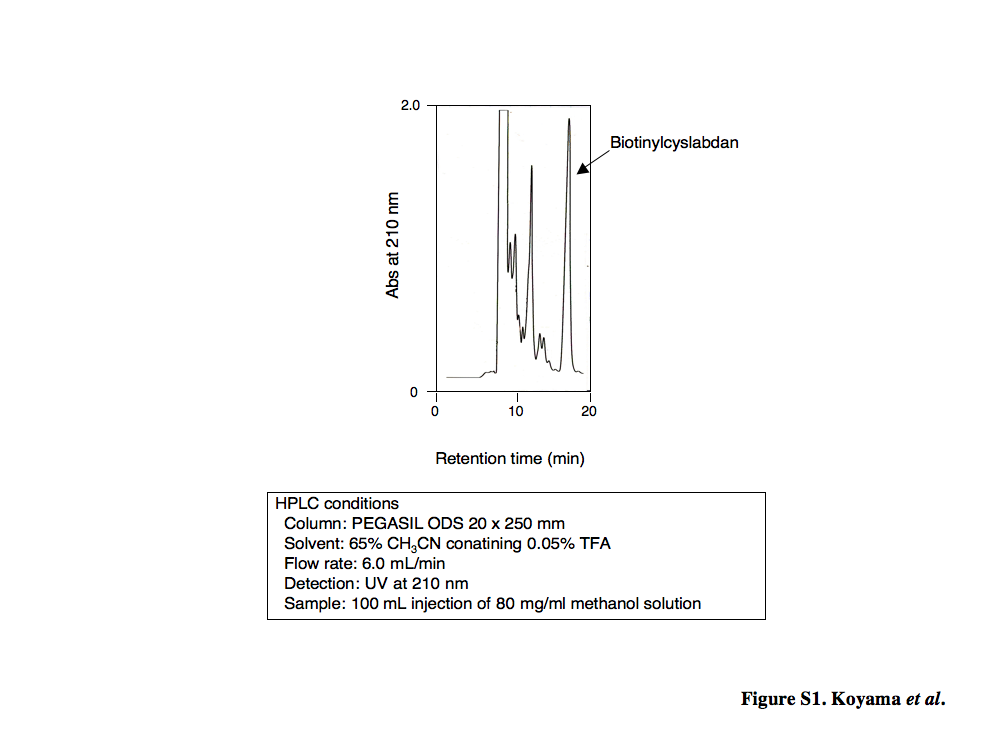

Supplement: Figure S1 — Purification of biotinylcyslabdan via HPLC. (TIF) [file pone.0048981.s001.tif]

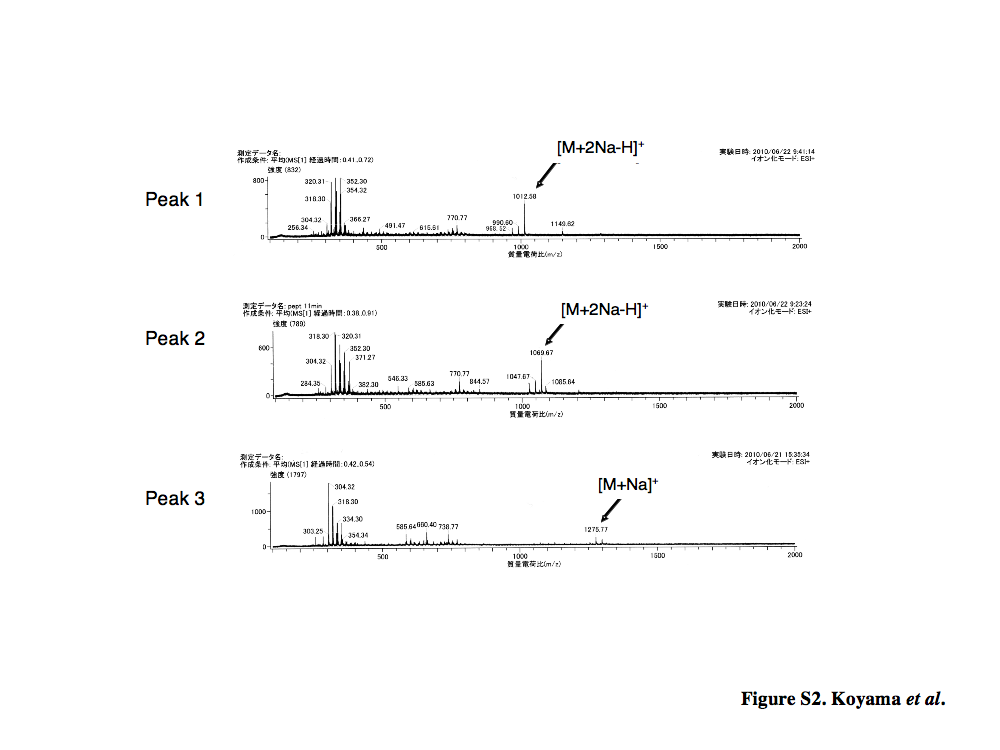

Supplement: Figure S2 — ESI-MS spectrum of the muropeptides identified in the monomeric region of the cell wall of MRSA. (TIF) [file pone.0048981.s002.tif]

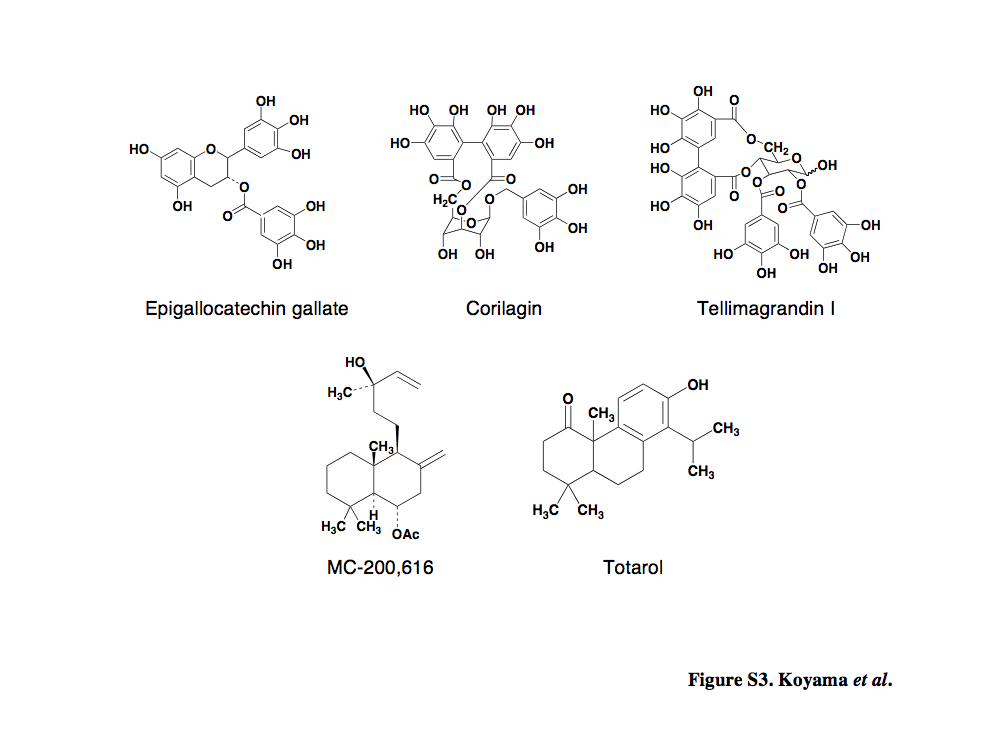

Supplement: Figure S3 — Structures of reported imipenem potentiators. (TIF) [file pone.0048981.s003.tif]

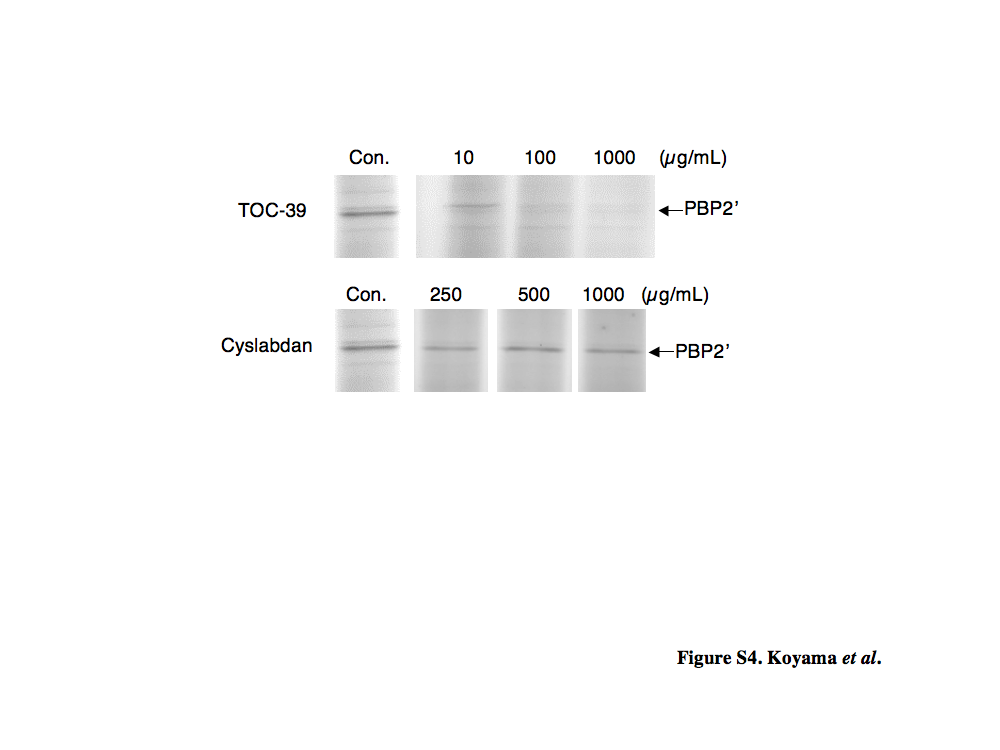

Supplement: Figure S4 — Effect of cyslabdan on PBP2′ binding in MRSA. An MRSA lysate was preincubated with clavulanic acid to mask PBPs other than PBP2′. The sample was treated with TOC-39 (10–1,000 µg/mL, upper panel) or cyslabdan (250–1,000 µg/mL, lower panel), followed by the labeling of PBP2′ with the fluorescent penicillin BOCILLIN. The samples were analyzed by SDS–PAGE. The arrow indicates the position of PBP2′. (TIF) [file pone.0048981.s004.tif]

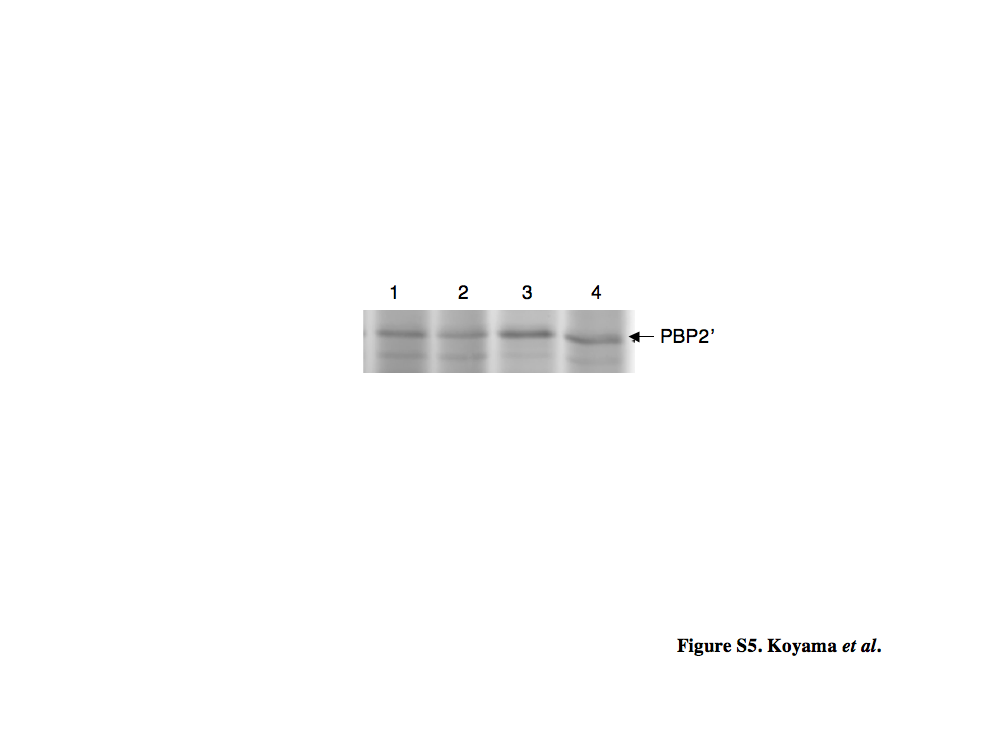

Supplement: Figure S5 — Effect of cyslabdan on PBP2′ expression in MRSA. PBP2′ proteins were extracted from MRSA treated with methanol solvent (lane 1), imipenem alone (lane 2), cyslabdan alone (lane 3), and cyslabdan plus imipenem (lane 4) and analyzed via SDS–PAGE using BOCILLIN. Concomitantly, the remaining sample was used to perform a latex agglutination test using an anti-PBP2′ antibody. All samples exhibited the same titer of agglutination (×1/32). (TIF) [file pone.0048981.s005.tif]

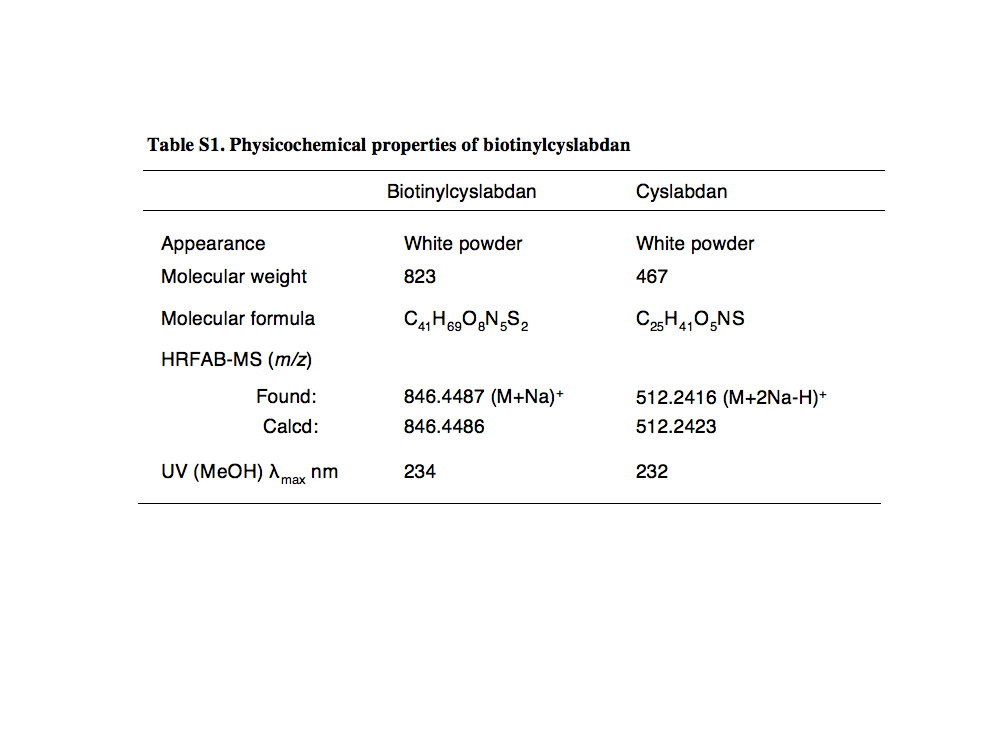

Supplement: Table S1 — Physicochemical properties of biotinylcyslabdan. (TIF) [file pone.0048981.s006.tif]

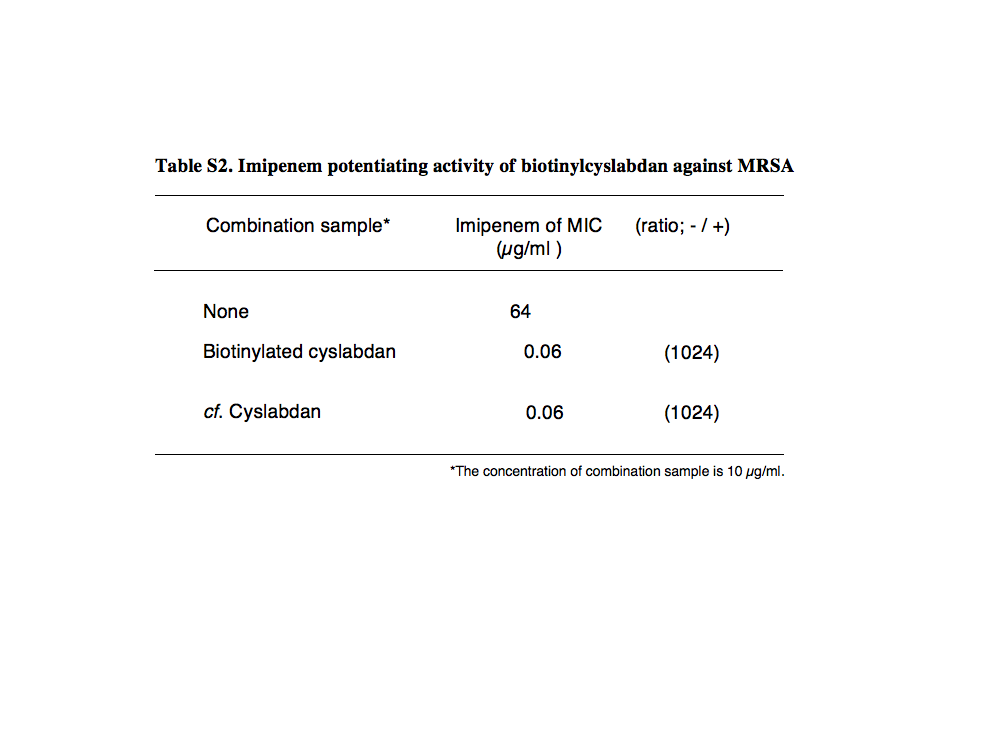

Supplement: Table S2 — Imipenem-potentiating activity of biotinylcyslabdan against MRSA. (TIF) [file pone.0048981.s007.tif]
